# Radiationless optical modes in metasurfaces: recent progress and applications

**DOI:** 10.1038/s41377-024-01548-5

**Published:** 2024-08-16

**Authors:** Naseer Muhammad, Zhaoxian Su, Qiang Jiang, Yongtian Wang, Lingling Huang

**Affiliations:** 1 School of Optics and Photonics, Beijing Engineering Research Center of Mixed Reality and Advanced Display, Beijing Institute of Technology, Beijing 100081, China, Beijing, 100081 China; 2 Beijing Engineering Research Center of Mixed Reality and Advanced Display, Beijing Institute of Technology, Beijing, 100081 China

**Keywords:** Optics and photonics, Applied optics

## Abstract

Non-radiative optical modes attracted enormous attention in optics due to strong light confinement and giant Q-factor at its spectral position. The destructive interference of multipoles leads to zero net-radiation and strong field trapping. Such radiationless states disappear in the far-field, localize enhanced near-field and can be excited in nano-structures. On the other hand, the optical modes turn out to be completely confined due to no losses at discrete point in the radiation continuum, such states result in infinite Q-factor and lifetime. The radiationless states provide a suitable platform for enhanced light matter interaction, lasing, and boost nonlinear processes at the state regime. These modes are widely investigated in different material configurations for various applications in both linear and nonlinear metasurfaces which are briefly discussed in this review.

## Introduction

Metasurfaces are a version of bulk-metamaterials arranged in two-dimensional manner possesses incredible capability of light manipulation, emission and localization^1–7^. Metasurfaces have been extensively recognized for potential applications^8,9^ in both linear and non-linear systems including sensing^10–12^, control^13^, modulation^14^, holograms^15–20^, optics for artificial intelligence^21^, meta-lenses^1,22–26^, light emission^27,28^, and lasing^29–31^. Metasurfaces can be subdivided by their materials configurations such as plasmonic metasurfaces^32^, dielectric^33–37^, transition metal dichalcogenides (TMDs) based^38–40^, and hybrid metasurfaces^41–44^. *Plasmonic metasurface* is a widely investigated area of nanoscale optics for a broad range of applications. The plasmonic nanostructures are made of metals and known for their well-pronounced optical signatures. The main bottleneck of plasmonic metasurfaces is the radiative losses in metals and noble metals in particular. Due to lossy nature of metals the plasmonic based devices usually show low Q-factor and nonlinearity which hinders some interesting applications^36,45^. *Dielectric metasurfaces* emerged as a low-loss alternative to plasmonic metasurfaces which basically comprised of high-refractive index materials^20,26,46–48^. Dielectric structures are investigated to replace or outperform the plasmonic nanostructure in some applications because of their high Q-factor and strong field confinement (both magnetic and electric) capability^46^. *TMDs based metasurfaces* is a newly emerged field usually made of few-layers ultra-thin geometric configurations^38,39^. Few layers TMDs possesses high refractive index in visible and part of infrared waveband, high optical density, and strong Columb interaction compare to common dielectric nanostructures in ultra-thin geometries. Though, to handle with radiative losses and further the overall efficiency of metasurfaces in infrared waveband a synergetic combinations of different materials have been reported as a *multiphysics or hybrid metasurface*^41,43^.

In the past, various optical features have been investigated in metasurfaces such as electromagnetically induced transparency and Fano resonances with high Q-factor^2,37,49,50^. Fano resonance is an asymmetric line-shape emerges from the coupling of a bright or radiative mode and dark or non-radiative discrete mode^49,51^. The radiative state can be directly excited by shining the nanostructure with incident electromagnetic beam. The broad mode results in low Q-factor optical features. However, the dark mode having high Q-factor can be thrilled by breaking the symmetry^36,39,52^ of the nanostructures or placing a number of nanostructures in a single unit-cell^45^, to mention a few. In recent past, detection of radiationless modes took the field of metasurfaces by storm and spreading their army to various applications. One of the non-radiative modes is *bound-states in the continuum* (BIC) which possesses infinite Q-factor and complete mode confinement^53–55^. BIC is a discrete mode in radiative continuum with zero-net radiation. Various mechanisms have been used to completely confine the optical state such as symmetry protected or separability, tuning parameters (coupled resonances and single-resonance parametric BIC), and BICs from inverse construction (hopping rate, potential and boundary shape engineering)^55^. In metasurfaces symmetry protected and accidental BICs have been exploited. Symmetry protected BIC in periodic structures (in x, y periodicity) is realized, when the rotational symmetry C_2_ around Brillouin point is preserved and x-, y-components of emission wave-vector are zero in that scenario these states are decoupled from radiation and leads to infinite lifetime and Q-factor^30,53^. In accidental or Friedrich-Wintgen BIC the interference of radiation from the two resonances with different frequencies and radiation rates which radiates to the same channel, result in one eigen values in to real and leads to BIC and other turns to more lossy^55^.

BICs have been exploited in different nanophotonics subwavelength configurations using metal^56^, dielectric^53,57,58^, TMDs^39^, perovskites^28,59,60^ and hybrid structures^61^. In ideal cases, BIC can be transform to quasi-BIC (q-BIC) via reducing symmetry of the structures, incorporating losses in the materials, and by varying the angle of applied incident electromagnetic beam. In practical cases, the BIC can be reduced to q-BIC by placing the BIC-inspired suspended nanoparticle on a substrate material^62^. The q-BIC results in a giant finite Q-factor and lifetime because of introducing a radiation channel. The interesting feature of BIC is to achieve wanted Q-factor and radiation by controlling the transformation parameters.

Another non-radiative or less-radiative mode is *anapole mode*, is the destructive interference of electric and toroidal dipole (resemble to the current flow on surface of torus) which results in vanishing scattering and accompanied by non-trivial distribution and confinement of field^38,63,64^. Toroidal multipoles are of two kinds, magnetic polar dipole and electric polar dipole. Along the ring surface or a torus, a polarized current flow forming closed loop arranged in head-to-tail manner in the magnetic field profile construct an electric toroidal dipole (ETD). In the same way, when poloidal electric current is flowing along the meridian of the torus and forming head to tail loop which can be observed from electric dipole or electric field profile is the magnetic toroidal dipole (MTD)^65,66^. Anapole mode has been reported in various material configurations using metals^63^, dielectric^64,67^, and TMDs^38^. Anapole modes are generally low Q-factor modes compare to BIC modes. Contrary to the dark modes in plasmonic nanostructure (due to symmetry it cannot be easily excited) anapole mode can be directly excited with planer incident beam at a certain aspect ratio^68–70^. Such radiationless (or weakly radiative) modes hold incredible potential of applications in the field of metasurfaces and optics which drive this review.

Previously, Hsu et al., have discussed the basic physics and mechanism of BICs. Each kind of BIC has been explained; provide basic understanding and a great collection of various types of BICs^55^. Baryshnikova, et al. reviewed the basic concept, physics and applications of anapole modes^71^. Koshelev, et al., discussed the BICs in isolated structures or single nanoparticles and metasurfaces. In this mini-review article the basics and physics in both cases have been discussed^72^. In recent past, extensive progress has been made in both topics. Various new applications have been discussed. Different kinds of metasurfaces including plasmonic^41,56,59,73,74^, dielectric^58,75–81^, TMDs^39,82,83^, perovskite^28,59,60,84^ and multiphysics^41,85,86^ metasurfaces have been demonstrated in both linear and nonlinear systems. The previous review articles lack TMDs and perovskite based metasurfaces which are newly emerging directions. In addition, some topics and mechanisms including Brillouin Zone folding^87^, BIC and anapole in chiral structures^61,87–91^, tuning of emissions and Q-factor^92,93^, anapole enhanced SHG in TMD based metasurface^83^, lasing from perovskite and semiconductors at both cryogenic and room temperatures^30,81,92,94–96^, high harmonic generations^97,98^, dual-band polarization up-conversion photoluminescence and tuning the coupling-strength to achieve BIC and exceptional points^79,95^ to mention a few, have been reported.

In this review we discuss the recent progress and applications of radiationless modes in plasmonic, dielectric, TMD, perovskite and multiphysics-metasurfaces. BIC in plasmonic nanoparticles demonstrate low Q-factor due to losses and weak toroidal dipole in case of anapole modes. This gap is filled by dielectric nanostructures with high Q-factor in both ideal and practical cases of BIC, also the strong contribution of toroidal dipole to realize anapole in dielectric metasurfaces has been explored. In dielectric metasurface the two eigenmodes with intrinsic toroidal dipoles with opposite moments destructively interfere and give-rise a toroidal dipole BIC. BIC and anapole modes resulted from the combine effect of excitons and optical modes in TMD based metasurface have been exploited. The coupling of metasurfaces with other materials facilitates the multi-physics metasurfaces which is a new area, have been exploited for enhanced nonlinear efficiencies. The strong coupling of excitonic and polaritonic excitations with optical modes result in new regimes and phenomenon. Such hybrid multi-physics metasurfaces demonstrates distinct groups of high-Q states. We also discuss BIC and anapoles in nonlinear metasurfaces and list some applications. Metasurfaces have gained attention due to their diverse applications, witnessing a move to commercialization which can be understand from the investment of corporations and aspiring start-up companies in this field. Although, still this area facing engineering, physics and fabrication challenges which are briefly discussed at the end.

## Material systems to design non-radiative strong optical modes

### Plasmonic nanostructures

In plasmonic devices one needs to suppress both radiative and Ohmic losses which determine the Q-factor of the system^45,99^. The dissipation increases with decrease in effective mode volume because the tighter is the confinement of field at the surface of metal, the fraction of modal energy is higher inside the metal^100^. This light-plasmon interaction inherent lossy feature affect the applications, however, some strategies like placing gain material in close proximity to the metal surface have been used to deal with losses^101^. The enhanced electric field due to plasmon excitation in the resonators can modify the radiative and non-radiative properties which lead to loss free operation in judiciously engineered configurations^49,99,100,102,103^.

**Fig. 1:**
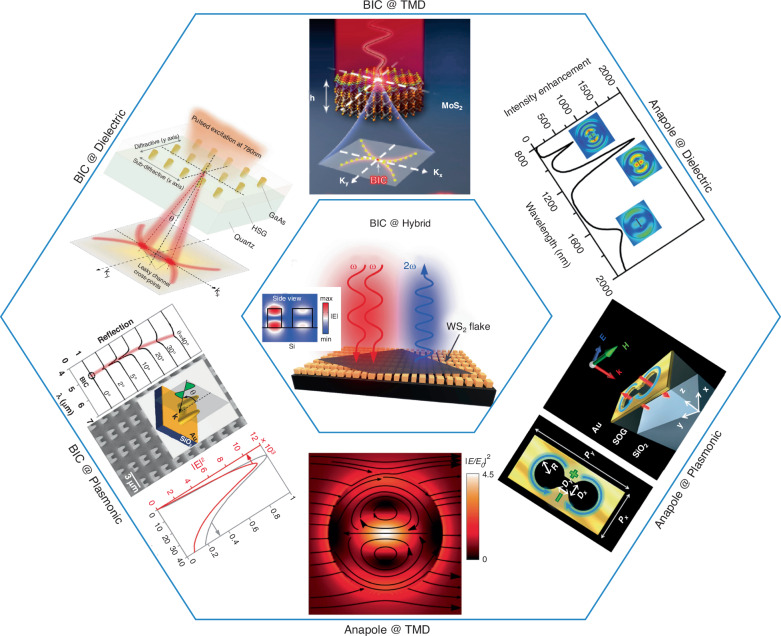
Recent advances in BIC and Anapole states in TMDs, all-dielectric, plasmonic and hybrid metasurfaces. BIC in TMDs metasurface^39^. Reproduced with permissions^39^. Copyright © 2021, American Chemical Society. K-space emission and lasing from BIC hosted dielectric structure^30^. Reproduced with permissions^30^. Copyright © 2018, Springer Nature Limited. Near field intensity in plasmonic structure based BIC^56^. Reproduced with permissions^56^. Copyright © 2020, American Chemical Society. Anapole mode in TMDs^38^. Reproduced with permissions^38^. Copyright © 2019, Springer Nature Limited. Plasmonic based metasurface^63^. Reproduced with permissions^63^. Copyright © 2018, American Chemical Society. Dielectric metasurface^35^. Reproduced with permissions^35^. Copyright © 2018, American Chemical Society. Second harmonic generation from WS_2_ coupled to dielectric (hybrid) q-BIC structure^85^. Adapted with permissions^85^. Copyright © 2020, American Chemical Society

The ideal case of BIC is reported in metallic structure with Q-factor up-to infinity. However, these losses puncture the Q-factor of *q*-BIC in plasmonic systems^56,74,104^ and does not meet the narrative of giant and wanted Q-factor developed for q-BIC in metasurfaces in the past decade (BIC @ Plasmonic in Fig. 1). A quasi-planar metallic structure support dynamic anapole resulted by destructive interference of electric and toroidal dipole^63^ (Anapole @ Plasmonic in Fig. 1). The Ohmic resistance of resonators restricts the excitation of toroidal multipoles due to their weak coupling to the external beams. The intrinsic losses in metals and low-damage threshold restrict the plasmonic devices for both linear and nonlinear applications. Plasmonic metamaterials have been used to enhance the nonlinear effects and control the polarization of light. In ordinary optical media the nonlinear effects arise from the electric field, although some cases in plasmonics entirely support it from the magnetic field^105^. In plasmonics, the excitation is highly sensitive to small perturb in the surrounding media and dielectric properties of metals. This is the main base for sensing and detection to monitor the small changes in the refractive index near the metal surface^10,73,106–109^. This provides a possibility to tailor dispersion and optical features by controlling the structure configuration and nature of surrounding material. Thus, the optical signatures can be tuned to the desired wavelengths for linear and to the position where nonlinear response wanted to be enhanced. For nonlinear nanophotonics, a control field can induce nonlinear variations in dielectric properties of material which leads to change in resonances and propagation of beam, thus control light by light. Raman process can be enhanced with plasmon excitation by orders of magnitude to detect a single molecule^110–113^. Similarly, enhanced third harmonic generation has been reported in nonlinear plasmonic metasurface^114^. Also, plasmonic excitation allows ultrafast processing of optical signals due to its response to few femtoseconds on timescale and leads to interesting nonlinear nanophotonics functionalities^103,115^.

### Dielectric nanostructures

The pursuit for using dielectric materials as an elementary unit in high-Q optics has established a trend in nanophotonics. The aim of using dielectric nanostructures is to circumvent the challenge of losses and heating in metallic structures. Owing to confined Mie type resonances they maintain, the high index materials exhibit comparatively strong interaction with light and acting as a resonant optical nanoantennas. Strong field confinement in low-loss dielectric resonators provided a route to optically induced magnetic response with magnetic-field multipoles^37,116,117^. Recently, all-dielectric meta-atoms have been used to confine partial and complete modes for various applications.

BICs have been exploited in various nanophotonics subwavelength resonators using silicon^52,78,118–120^, gallium arsenide^30^ (BIC @ Dielectric in Fig. 1), silicon nitride^121,122^, indium gallium arsenide phosphide^29^, and aluminum gallium arsenide^123,124^. BIC has been realized due to destructive interference of two eigenmodes with toroidal moments along y and z-axis which reveal link between toroidal dipole and BIC. The two eigen-modes in dielectric disk-dimer show intrinsic dipolar character of toroidal modes with infinite Q-factor and lifetime^125^. To eliminate the major bottlenecks of toroidal metadevices the anapole state was reported experimentally for the first time in silicon disk and the states were detected by varying the geometric parameters of the disk in visible range^64^. Dielectric support both electric and magnetic modes and is suitable platform for a kind of magnetic modes^35^ (Anapole @ Dielectric in Fig. 1). The dielectric can outperform the metallic structures in nonlinear regime^126^ due to inherently low nonlinear response in plasmonic materials. The dielectric possesses low losses and maintains much strong optical power which ultimately leads to orders of magnitude high frequency conversion. The nonlinear efficiency conversion can be further boosted by excitations of both magnetic and electric nature multipoles.

### TMDs

TMDs layered materials in both monolayer and bulk configurations have been used for various optoelectronic applications^127–129^. The atoms are strongly bonded in the same plane but weakly attached with up and down layers by van der Waals forces. The weak van der Waals forces and the layered nature make the layer exfoliation from bulk to monolayer possible, and resulted in a broad research area of two dimensional materials for photonic applications^130,131^. As the thickness decreases from few layers to single layer, the bandgap modulate from indirect to direct providing windows to various optical applications. The monolayer of MoS_2_ provides the large nonlinear optical coefficient and extraordinary contrasting physics when the inversion-symmetry is reduced unlike their bulk counterparts^132^. TMD materials have high background dielectric constant which is higher than semiconductors such as germanium and silicon. In common dielectric the dielectric constant depends on the band structures, however, in TMDs it is dominated by the tightly bound excitons. A and B-excitons amplitude in WS_2_ is independent of number of layers and arises from the spin orbit-splitting and inter-layer interaction. A Fabry-Perot mode appears close to exciton energy and hybridizes with excitons at about 70 nm thickness of the multi-layer flakes due to their high background refractive index. So, the TMD material supports FP cavity and exciton which couples and further result in self-hybridized modes without any external cavity^133^.

Due to high optical density and strong Columb interaction in ultra-thin geometries, excitons and optical modes in TMD based metasurface team-up to originate BIC^39^ (BIC @ TMD in Fig. 1). Monolayer TMDs coupled with dielectric materials have been reported for enhanced nonlinear efficiencies in BIC hosting configurations^85,134–138^. The coupling of BIC hosting photonic structure with a monolayer TMD is deposited at the top of dielectric structures (BIC @ Hybrid in Fig. 1). However, the transverse electric modes are localized within the dielectric structures which result in weak exciton-photon coupling. In Bloch surface waves the electric field enhancement is confined at the surface allowing the strong coupling of the resonant modes in TMDs^138,139^. These approaches can introduce the in-homogeneities while depositing monolayer TMDs which can result in excitons spectral shift and nonlinear effect suppression due to strain and topographic irregularities in TMD layers^140,141^.

Similarly, anapole mode has been demonstrated in isolated nanostructure^38^ (Anapole @ TMD in Fig. 1). TMDs are known for their strong light-matter interactions and extraordinary valley-contrasting physics. From statistics high quality monolayer TMDs coupled with dielectric or plasmonic nano-holes can produce enhanced nonlinearity compared to bulk counterparts^15,85^. However, a carefully engineered unit-cell of optimized nonlinear bulk TMD can produce a strong second harmonic generation (SHG) signal^40,132^.

### Multiphysics

**Fig. 2:**
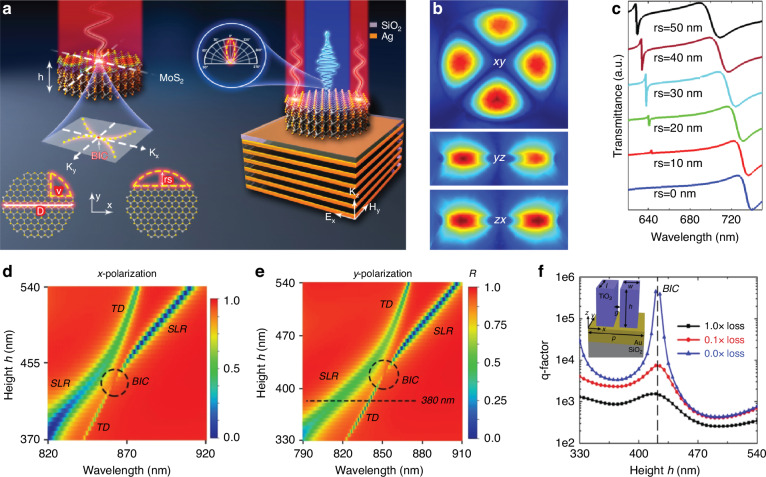
BIC in few-layers TMD based metasurface and dielectric-metal-dielectric stacked structure. **a** Schematic of TMD based metasurface^39^. **b** Magnetic field profiles of q-BIC state at corresponding wavelengths^39^, **c** Transmission spectra at different symmetry breaking cuts^39^. Reproduced with permissions^39^. Copyright © 2021, American Chemical Society. **d**, **e** Reflection spectra for dimer height h as a function of wavelengths under x- and y-polarized normal incidence. The formation of BIC is highlighted by a circle in two indicated branches of states^61^. **f** The q-factor a function of dimer height *h* for the quasi-BIC mode with different incorporated losses^61^. Adapted with permissions^61^. Copyright © 2020, American Chemical Society

The hybrid combination result in BIC with giant Q-factor (Fig. 2d–f) when the losses are reduced by tuning the imaginary part of the refractive index. Due to synchronized excitation of q-BIC in both x- and y-polarization cases the well-matched field distribution benefitted in strong-chiral fields^61^. In plasmonic system high-Q BIC realization is a challenge due to high losses. To eliminate the losses and achieve high Q-factor surface plasmon polaritons have been coupled to photonic modes in a synergetic arrangement of plasmonic-metallic system. Two different kind of BICs have been realized in plasmonic-photonic system due to suppressed radiation and strong mode coupling of 150 meV^41^. In one dimensional layered photonic crystal Friedrich Wintgen BIC cannot be realized due to separated polarization. It can be realized because of destructive interference with different polarization using defect layer which mixes the light polarizations. Pankin et al., demonstrate BIC in 1D photonic crystal with anisotropic defect layer covered by metal film which plays role as a quantum well^142^. The toroidal mode which contributes to realization of anapole mode can be excited strongly in homogeneous dielectric materials compare to metallic ones. The hybrid combination of metallic and dielectric materials support trivial (without multipoles excitation) and non-trivial (with multipoles excitation and destructive interference of toroidal and electric dipole) transparencies^143^. A combination of dielectric-metal-TMDs has been reported to demonstrate anapole-plasmon and anapole-exiton-plasmon coupling at normal incident field, which open-up a new window for multiphysics devices^86,144^.

## Approaches for BIC

In quantum system, the quasi-stationary modes are responsible for the resonances in the scattering peaks. In 1929, von Neumann and Winger reported the bound-states in quantum system above the continuum-threshold^145^. In 1992, Capasso et al., demonstrated it in one-dimensional physical system and thereafter, it was verified that BIC state holds infinite lifetime and occur due to quasi-stationary states interaction^146^. The exception of BIC is coexisting with extended waves inside the continuum with no radiation due to no-pathway to radiate out. Contrariwise, a mode inside the continuum with radiation out to infinity can be a resonance or leaky state^55^.

Similarly, metasurfaces as a periodic arrays have long-lived confined field states which support enhanced reflectance/transmittance or both. In the past decade, BIC has been reported in subwavelength single (isolated) nanoparticles^34^ and metasurfaces^39,61,147,148^ periodic in both *x* and *y* or in one- direction. Kim et al., discussed symmetry protected BIC at Г point and symmetry is reduced with non-zero axial wave-vector. With distorted illumination symmetry and deviation of wave-vector from Г point the Q-factor decreases. The 1D structure also supports a BIC when the size of inner diameter is increased at fixed outer diameter of the geometric super-lattice. The BIC is formed by the complete destructive interference of modes accumulated by the geometric super-lattice. Experimental results meet the theoretical calculation and BIC appearance with variations in geometric parameters. It is shown that the H_y_ and E_y_ components verified by the eigenmodes analysis reveals that the Q-factor of the modes supported by the nanowire is diverged to infinity at a discrete position with infinite lifetime^148^. Yang et al., demonstrate off-Г BIC in photonic crystal slab, and can be tuned by variation in wave-vector^121^. Rybin et al., studied BIC and supercavity modes in subwavelength homogeneous high-index single nanoparticle. The BIC is formed due to the strong interaction of eigenmodes with different polarization.

The cylinder-shaped finite length resonator illustrates modes associated with Mie-like and Fabry-Perot resonances. Both modes are nearly orthogonal inside and their predominant interference realizing BIC and supercavity mode when it establishes high Q-factor. Due to different spectral shifts of Mie and Fabry-Perot resonances they intersect each other and interact at the same azimuthal index. The modes coupling occur with avoid-crossing scenario at certain vales of aspect ratio and the spectral line approximately disappears near the supercavity mode^34^. Plotnik et al., experimentally demonstrated the existence of BIC in an array system. They reduced the symmetry of the structure with adding refractive index gradient gradually to manifest the light escape and coupling with continuum^149^. Molina et al., discussed the concept of surface BIC modes existence in the waveband and tunability with adding nonlinearity^150^. Zhen et al., presented the topological nature of BIC realized in photonic slab. They revealed that in the far-field radiation BIC is the vortex center carrying quantized and conserved topological charges. The strength and robustness of BIC is defined by the number of polarization vectors. Large number of vortex needs strong disturbance to transform BIC to a q-BIC^53^ because the topological charge at the center of the nodal lines (E_x_ and E_y_) and the wave-vector components (k_x_ and k_y_) become zero. In this scenario the precise dipole-oscillation (with no radiation) in normal direction and destructive interference of the other dipoles in all other directions in the structure leads to zero-radiation^30^. A synchronized crossing at zero yields in no outgoing power; consequently the mode is completely confined. If the k_x_ and k_y_ are varied then the outgoing power is unlikely to be zero^151^. Such simultaneous crossings are distinct from the leakage disappearance which is due to destructive interference amongst several radiation channels. The field profile in the photonic slab is the superposition waves having different propagation constants in the in the propagation direction. Partially, each wave is reflected back and a part is transmitted by the photonic slab medium interface which turns out to be the outgoing plane-wave. At an appropriate k points the transmitted waves with different propagation constants may cancel each other and result in destructive interference.

Cong and Singh reported symmetry protected dual BIC in a subwavelength planar metamaterials structure. The BIC is observed by breaking the C_2_ symmetry to open the radiation channel at the discrete position^147^. Xu et al., reported BIC in a thin silicon-based disk, the structure supported BIC mode at 53 nm thickness. The symmetry breaking of the disk-shaped structure invoke the leakage channels^52^. Gurkonov et al., discussed chirality empowered by BIC in a bar dimer structure. The BIC transformation to *q*-BIC is controlled by offsetting the structure and incident angle of applied electromagnetic beam^152^. Benalsazar and Cerjan, demonstrate corner localized BIC in lattices with higher order topology^153^. Lee et al., reported continuous high-Q BIC in metasurface in a broad-range of wave-vector instead of a discrete one^154^. BIC is achieved in low refractive index resonator on the top of high refractive index substrate an integrated photonic structure^80^. Anisotropic induced BIC with new properties was discussed in a waveguide structure contain birefringent materials^155^. A photonic crystal structure supports the coupling of directive BIC to effective near-zero-index to suppress the radiation losses^156^.

Naseer et al., reported a BIC in TMD based metasurface with ultra-thin geometry (Fig. 2a–c). The BIC mode is supported by the high refractive index and exciton effect. By breaking symmetry of the disk the structure maintain the spectacular shape of localized modes which reveal the tunability of the TMD materials^39^. Recently, Brillouin zone folding has been applied to engineer modes into BIC at the edge of first Brillouin zone^28,157–163^. BIC engineered with Brillouin zone folding shows dramatic increase in Q-factor with disorder in the space between holes in the photonic crystal slab in the momentum space and immune to changes in structure^76^. BICs and exceptional points can be achieved by tailoring the spectral features and changing the coupling strength from weak to strong^79^.

In real system the losses play role to transform the BIC to a q-BIC or a resonance with finite Q-factor. This occurs due to the leakage in substrate which suppresses the BIC mode to a resonance with finite lifetime. From both theoretical and experimental analysis it is observed that the BIC transformation to a resonance is due to the surface roughness, thickness of high-index substrate, and penetration of near-field to the substrate^62^. Kühne et al., demonstrate both theoretically and experimentally the effects of dimensional variations on performance of BICs in different configurations^164^. In plasmonic systems BIC can be achieved for ideal cases although, due to strong associated losses the practical case of BIC-inspired structure results in low Q-factor^56^. To avoid the quick drop of the Q-factor in q-BIC one can use a hybrid combination made of plasmonic-photonic synergetic arrangement^41^. The careful arrangement leads to the coupling of photonic modes to the plasmonic ones and can open-up a new window for various applications in the field of optoelectronics.

The Q-factor of BIC hosting device is low due to structure disorder, roughness and variation in structure fabrication. In practical case the Q-factor is comprises of non-radiative and radiative parts, where the non-radiative part covers the fabrication imperfections. The low non-radiative part in particular, results in low Q-factor of the meta-atoms and therefore, hinders the applications. To achieve high Q-factor experimentally, different sizes of metasurface arrays were fabricated consisting on monocrystalline silicon with various defect sizes on a quartz substrate as reported in^165^. They used two driving parameters to reduce the line-width of q-BIC and enhance the Q-factor. First they fabricated the metasurface consists of 21 × 21 unit cells with different defect sizes. The metasurface results in sharp spectral signatures of q-BIC having high Q-factor and well-fitted with simulations. Next, they fabricated the metasurface with different array sizes, and it is demonstrated that the Q-factor increases with increase in number of unit-cells. Such high-Q resonances are appealing for various nanophotonic applications, in particular, reducing cross talk, bio-sensing and slow-waves.

## Anapole modes

Compared to high-Q BIC modes anapole is usually low-Q non-radiative state. Anapole modes appear due to destructive interference of electric and toroidal dipoles when these modes are tuned to become out-of-phase. Electric dipole is linear oscillation of currents that induce rotating magnetic field. Since there are no magnetic charges, although, the magnetic and electric vector field symmetrically enters the Maxwell’s equations. The toroidal electric current generate a loop supporting an effective magnetic current and result in same far-field pattern as electric dipole. The electric and toroidal dipoles cancel each other in the far-field region when both are out-of-phase and results in anapole state^81^. It has been reported that the ideal non-radiative anapole can be excited by radially polarized two counter propagating fields of the same amplitude and π-phase difference when the reciprocity condition is not violated^166^. However, the anapole is not an embedded eigen-mode and it is a field distribution at zero scattering which cannot be maintained without the incident excitation beam^167^.

The toroidal produce the identical radiation pattern in far-field as corresponding electric and magnetic multipoles. These multipoles provide significant input physically to the optical characteristics including absorption, optical cavity, and dispersion. The destructive interference of electric and toroidal dipole results in vanishing scattering and accompanied by enhancement of near-field^71,168^. Thanks to the identical radiation pattern when both are co-excited, the spatial overlap of out-of-phase radiation with same magnitude cancel the scattering effect of each other. Due to the destructive interference the scattering pattern disappears in the far-field region and leads to a non-radiative state with non-trivial oscillating current profiles^63,64^. Toroidal dipole induced transparency has been reported in non-radiating states in hybrid nano-sphere^143^. The nanostructure has enough freedom to shift the cancellation point of electric and toroidal dipoles to the position of magnetic dipole and quadrupole where both are highly suppressed to minimum scattering; therefore, the structure resulted in non-trivial toroidal dipole induced transparency. To achieve the non-trivial transparency the excitation of other multipoles must be suppressed. The effective excitation and destructive interference of toroidal and electric dipoles are the main conditions to be satisfied. Secondly the total scattering must be negligible due to destructive interference in scattering particle. The trivial transparency window is obtained in different structure which is resulted by not properly exciting the electric and toroidal dipoles compare to the former case. Hence, the hybrid anapoles demonstrate less coupling with the neighboring unit cells. Consequently, the devices can operate individually in highly dense arrangement which makes them robust and able to preserve the characteristics in the presence of dielectric substrates in contrast to Huygens particles ascribed to inter-particle coupling.

**Fig. 3:**
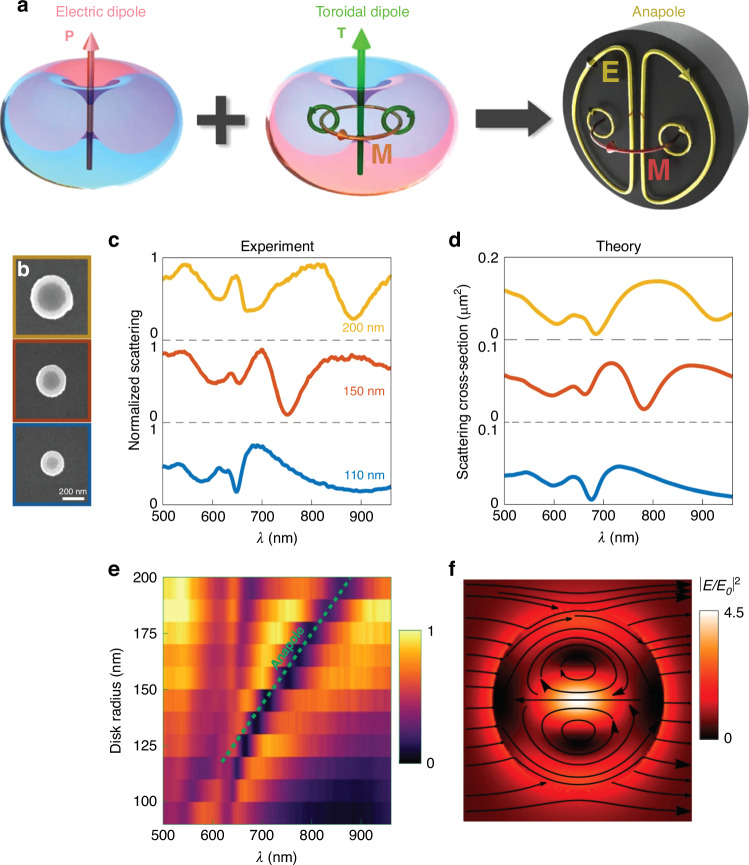
The mechanism of anapole in dielectric metasurface and demonstration in TMD metasurface. **a** Illustration of anapole excitation (toroidal dipole linked with circulating magnetic field accompanied by electric dipole). The identical radiation patterns of toroidal and electric dipoles can destructive interference results in total scattering cancellation^64^. Reproduced with permissions^64^. Copyright © 2015, Springer Nature Limited. **b** Experimental observation of anapole mode in WS_2_ resonator of different diameters^38^. **c**, **d** Experimental and theoretical normalized scattering spectra^38^, **e** Normalized dark-field scattering map for WS_2_ nanodisks (experimental) of disk radius vs wavelength. The spectra show a linear dispersion attributed to anapole mode as indicated by green dotted line^38^. **f** The electric field distribution profile shows an anapole-like feature, calculated theoretically and normalized to the free space^38^. Reproduced with permissions^38^. Copyright © 2019, Springer Nature Limited

In optics, anapole modes were discussed for the first time in thin metal-plate arranged in meridianal cross-section making a dumbbell-shaped metamaterials. The coherent oscillation of the electric and toroidal and their destructive interference leads to a non-radiative charge-current configuration. The multipolar current state is effectively suppressed due to high rotational symmetric geometry of the microwave metamaterials^169^. The toroidal dipole is weak compared to electric dipole in metallic structures, although toroidal dipole can be strongly excited in high refractive index dielectric based structures. Wu et al., reported anapole states in quasi-plasmonic structure. The quasi-plasmonic structure is consisting of dumbbell-aperture and a split-ring shaped resonator. The anapole state and transvers toroidal moment was detected and experimentally demonstrated in the optical wavelengths. The pattern of radiation of both dipoles is identical and a careful arrangement results in destructive interference under the incident plan-wave. For the magnetic anapole (suppression of magnetic dipole scattering can be achieved for large values of parameters) is with strong near-field enhancement the energy is pushed outside resembles to plasmonic nanostructures and in case of electric anapole the energy distribution is different. The field was confined inside the structures at the nanoscale^170^. In 2015, the theoretical predictions of radiationless states were experimentally demonstrated in a thin silicon disk in visible waveband. The spectral overlap of the electric and toroidal modes, near-field mapping and dip in scattering cross-section (due to strong suppression in far-field) depicting anapole mode was achieved by tuning the geometric parameters. In the nano-disk the near-field enhancement is escorted by the existence of toroidal and electric dipole which offers radiationless anapole states (Fig. 3a)^64^.

Recently, TMDs represented a radical departure from the lossy metallic optics paving the way to more efficient control and manipulation at the subwavelength scale. In TMDs, anapole modes have been demonstrated theoretically and confirms experimentally in few-layers WS_2_. The structure was excited by linearly polarized incident field and the scattering was calculated at the upper hemisphere. It was shown that the simulations better meets the experimental confirmation and the complex optical features were obtained with multiple peaks and valleys (Fig. 3b–f). The multipole decomposition indicated that the structure is hosting an anapole-like mode. The coupling illustration of exciton with geometrical dispersive anapole is achieved by incorporating the artificial isotropic dielectric. In hypothetical losses case the anapole-like mode appears when the A-exciton transition is off. When the exciton transitions are turned on the anapole mode couples to exciton and result in two well-pronounced dips. The scattering of dark and bright states destructively interfere in the far-field at the anapole wavelength. The A-exciton are excited by the near-field (or the energy stored) coupling. It is shown that two dips appears in the scattering cross-section which exhibit anti-crossing behavior. One of the dip is due to the destructive interference of the far-field and the second one corresponds to A-exciton^38^.

In subwavelength bulk TMD flakes when the thickness is in 60–70 nm range a FP cavity appears and hybridizes with excitons^133^. The structure having artificial dielectric function without A-exciton leads to well-pronounced zero scattering anapole dip and with only A-exciton a scattering valley appears at exciton wavelengths. In the third case when refractive index and A-excitons both are on, the resonator yields three scattering minima. One of the dips is at the anapole wavelength and second at excitons positions due to hybridization. The third dip is because of excitons absorption which is not taking part in hybridization with Mie modes^38^.

Excitons can be coupled to plasmon and anapole state. The coupling of anapole mode, plasmonic modes and exciton in a layer of TMD has been demonstrated in the multiphysics system. It was noted that the generated polaritons produce strong Rabi splitting along with high field enhancement of 157 because of metal, low-scattering of anapole and high optical absorption of TMD^86^. Modes supported by both dielectric and excitons feature a plethora of exotic effects not reachable with dielectric and plasmonic; enable the ultrathin meta-devices for various linear and nonlinear optoelectronic applications.

## Nonlinear systems

Nonlinear optical effects contributes to concentrate light in subwavelength nanoscale to enhance light-matter interaction^115,126,165,171–173^, which allow the nonlinear processes such as all-optical switching^14,174,175^, wavefront control^176,177^, frequency conversion^4^, and nonlinear optical chirality^15^. At nanoscale, plasmonic effects have been extensively explored to control and enhance nonlinear processes including nonlinear imaging^178^, wave-mixing^179–181^, holography^182,183^, harmonic and super-continuum generation^4,114,184,185^. Although, dissipative losses and thermal heating result in low damage threshold and cannot withstand large pump field intensity^115^. The field confinement is limited to the surface of the metals. Compared to bulk counterparts the 2D materials monolayer TMDs provide large nonlinear optical coefficient and valley-contrasting physics. However, the optical absorption, field localization and domain size are the main bottlenecks which restrict wavefront control of nonlinear emission under common light sources^15,132^. The dielectric resonators support higher order magnetic resonances and survive at high pump field intensity^36,37^. However, the electric field confinement in dielectric resonators is weaker compared to plasmonic structures. Common dielectric such as Si and Ge possess centro-symmetry crystal structure due to which it can be utilized for enhanced third-order nonlinearities but not for second order^176,186^. However, amorphous silicon based metasurface has been used to demonstrate SHG. The SHG follows the selection rules and the signal is generated from the surface by increasing surface to volume ratio^187^. The group III-V semiconductors have large second order susceptibilities, dielectric constant and have been used for enhanced SHG in metasurfaces^179,188^. LiNbO_3_ and GaN can be utilized as a dielectric or main constituent due to its lower losses in the shorter wavelengths for second order nonlinear metasurfaces^189^. To enhance the nonlinear susceptibility of monolayer TMDs, Bernhardt et al., coupled it with a high Q-factor q-BIC inspired dielectric lattices. The dielectric lattices hosting BIC boosted the SHG by more than three orders compared to a TMD monolayer on the flat dielectric film with same thickness. The dielectric lattices originate the magnetic dipole moment and electric field is localized in the gap which boosts the field enhancement in monolayer TMD. It was shown that the structure results in 1140 times higher SHG in monolayer TMD coupled to metasurface with reduced symmetry compared to Si of the same height^85^. The interaction of non-radiative and radiative losses can manipulate the third-harmonic generation (THG) in dielectric structures. The metasurface can be tuned to critical coupling regime by tailoring the in-plane symmetry. When both losses (non-radiative and radiative at the intermediate value of asymmetry parameter) coincides a maximum frequency conversion efficiency can be achieved in BIC-inspired metasurfaces^126^. The interplay in nonlinearity, BIC, and higher order topology has been reported in a photonic lattice. They discussed the nonlinearity with edge states and transition to solitons at low and high nonlinearity respectively. They observed that the crystalline and chiral symmetries are broken by the nonlinearity albeit nonlinear coupling of BIC to the higher order states is weak^190^.

**Fig. 4:**
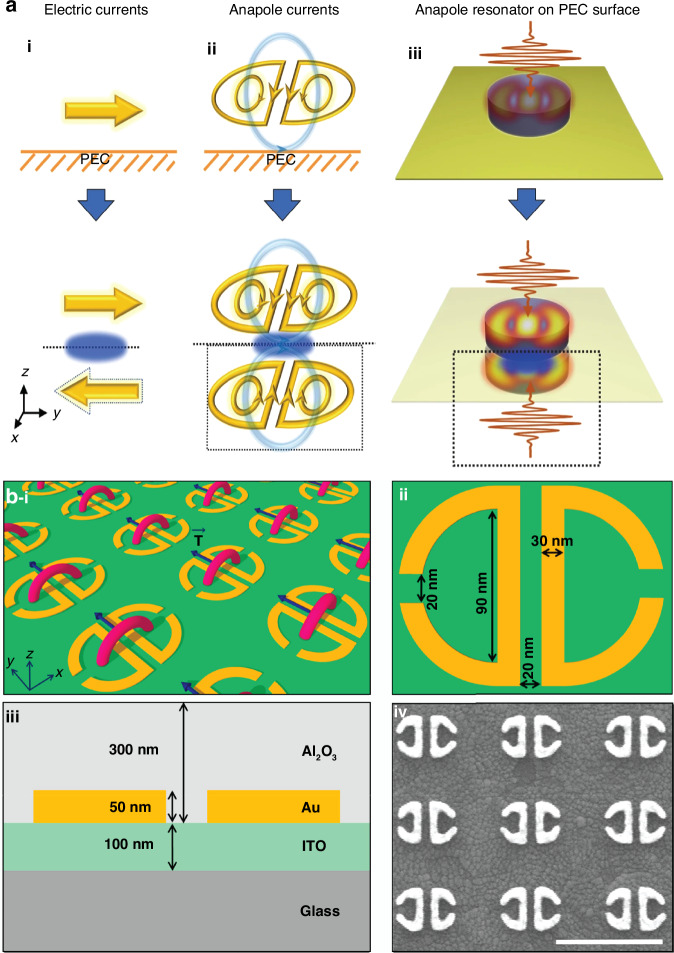
THG from anapole hosted plasmonic and dielectric metasurfaces. **a**–**i** Illustration of current excited in meta-atom on the top of perfect electric conductor substrate^168^ **ii**, **iii**. Anapole current and field distribution, this work facilitate boosting nonlinear efficiency^168^. Reproduced with permissions^168^. Copyright © 2018, Springer Nature Limited **b**–**i**. Direction of toroidal dipole moment and formation of charge current configuration in two resonators in plasmonic nonlinear metasurface^114^ **ii**–**iii**. Top view and side view^114^, and **iv**. SEM image of metasurface^114^. Reproduced with permissions^114^. Copyright © 2019, American Chemical Society

Zhang et al., discussed the active scattering tuning and giant photo-thermal nonlinearity in a dielectric nanodisk by using the near-field and far-field of anapole. Due to the strong absorption of near-field in nanodisk increase in temperature influences the refractive index of the structure with weak laser irradiance. A large variation in real and imaginary parts of refractive index with thermo-optic effect in visible waveband due to substantial temperature increase depends on the contribution of multipoles. It was shown that the position of anapole state is dynamically controlled and all-optical stimuli allow the active scattering modulation photo-thermally. The features of anapole modes have been manipulated desirably, thus resulting in nonlinear scattering responses^67^. Grinblat et al., achieved a valley in the scattering spectra characterizing anapole mode in high refractive index germanium^68^. Ospanova et al., presented anapole mode in a simple dielectric slab in visible waveband^191^. Sebastian et al., demonstrated the SHG from the bulk TMD materials. It was shown that second harmonic is low at thick resonator structures and can be improved with carefully engineered geometries^40^. The nanostructures with small or moderate refractive index can generate an anapole mode. Kim and Rim reported anapole state in LiNbO_3_ with moderate refractive index. It was shown that strong field enhancement and anapole states can be achieved in low refractive index resonator with using the near-zero index or metallic substrates^192^. It was reported that an anapole inspired structure placed on a metallic structure (see Fig. 4ai–iv) can enhance the THG emission two folds of magnitude compared to placing it on insulator^168^. Enhanced third harmonic generation from an indium tin-oxide film was significantly enhanced by metallic toroidal structure covered with alumina layer to avoid high threshold damage induced by laser. This was resulted from combining the high susceptibility of structure with field enhancement in toroidal hosted metasurface (Fig. 4bi–iv)^114^.

## Applications

### Bound-state in the continuum

#### Sensing

BIC has been investigated for various applications in metasurfaces due to strong field localization and high Q-factor. The q-BIC analogous to Fano resonances and electromagnetically induced transparency (EIT) with high Q-factor provides a suitable platform for detection and refractive index sensing^61,193^. Fano resonances are highly sensitive to the nature of background materials, and a small perturb can cause shift in optical features. The refractive index of the cancerous cells is expected to be higher than healthy cells due to high cellularity of tumors. However, a very small refractive index change needs high sensitivity of the resonances to detect minimum refractive index variation^193^. Chen et al., reported a sensitivity of 80 nm/RIU for BIC-hosting structure^61^. A sensitivity of 326 nm/RIU was reported in all-dielectric metasurface^75^. Although, the sensitivity and figure-of-merit values are very low compared to theoretically calculated in plasmonic metasurfaces^10^^,^^193^^,194^.

#### Image tuning

Quality enhancement of images is one of the applications of metasurface which is essential for various areas such as medicine and biology. Metasurfaces have been demonstrated for resolving features with small spacing, aberration-free optical images and magnetic resonance imaging for clinical diagnosis^195–197^. BIC dynamically control the nonlinear emission and can enhance the emission from the structure. It is demonstrated from the image tuning via THG process under different pump excitation polarization and wavelength position. Both images were encoded into metasurface, and the disks with different off-centered hole were excited with different polarization of pump. The geometric configuration tune the spectral position of BIC mode and the nonlinear image tuning have been achieved with wavelength tuning directly^52^.

#### Lasing

**Fig. 5:**
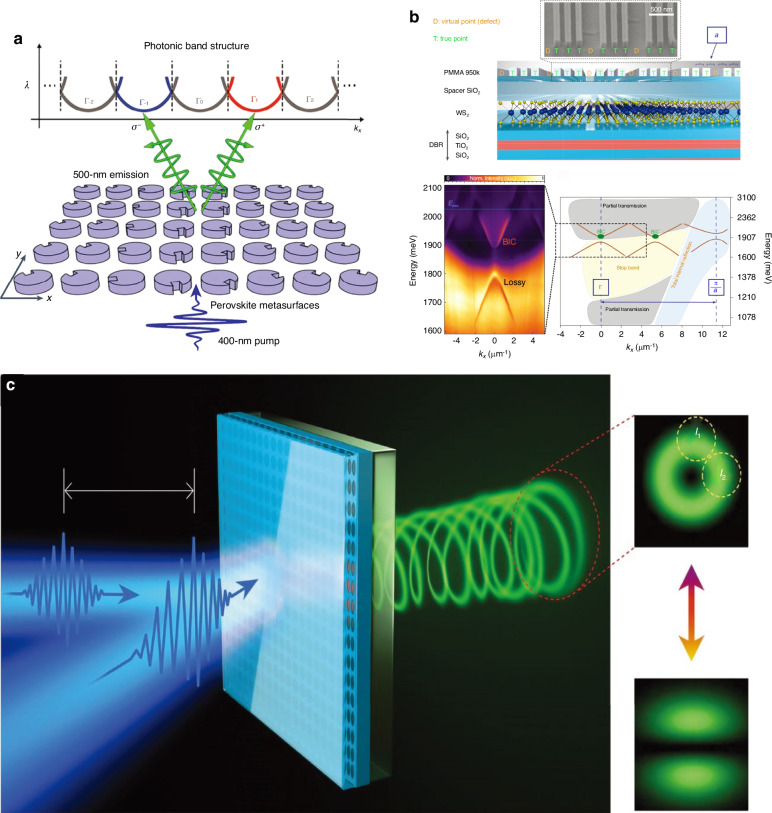
BIC in perovskite and TMD based metasurfaces. **a** Spin valley locked emission in perovskite metasurface^28^. Reproduced with permissions^28^. Copyright © 2023, Springer Nature Limited. **b** A monolayer TMD has been coupled with Bragg gratings to achieve 100 meV photonic bandgap and Rabi splitting of 70 meV in BIC inspired structure^138^. Reproduced with permissions^138^. Copyright © 2023, Springer Nature Limited. **c** Two beam pumping experiment for ultra-fast control of the quasi-BIC micro-laser. The insets are far-field emission pattern under symmetric and asymmetric excitation from the metasurface. With one applied beam the structure sustain the symmetry and result in a donut shaped and the normalized ratio is approximately zero. Applying the second beam reduce the symmetry. The metasurface switch vortex lasing to linearly polarized beam and back to vortex with switching time of 1.5 p.s^92^. Reproduced with permissions^92^. Copyright © 2020, The American Association for the Advancement of Scienc

The Q-factor of the vortex micro-lasers is usually reduced by scattering losses which leads to large energy consumption. The on-chip integrated vortex micro-lasers are controllable by circularly polarized optical pump in emission chirality, energy consumption and modulation speed causing limitation of their use in ultrafast optical networks. Laser emission from a BIC inspired metasurface can be all optically controlled because such lasers are extremely sensitive to a small perturb in variation in geometry. In symmetry protected BIC based laser the imaginary part of refractive index ∆n is used to control the symmetry and growth in ∆n transform BIC to q-BIC. The circular pump maintains the symmetry while the ellipse beam breaks the symmetry and produce two linearly diffracted beams. Similarly, the two applied circular beams one with above density threshold and other with below density threshold with 10 µm lateral shift can be used to break the symmetry. The lasing can be switched from vortex to linearly and polarized and back to vortex with a switching time of 1.5 ps^92^ (see Fig. 5c). In passive systems the symmetry transformation is realized by Kerr nonlinearity or distortion in the structures^198^ although, it requires strong optical excitations. Different orders of optical vortices can be achieved by scaling the structure and perturbing the symmetry^199^.

The suppression of losses and high Q-factor of BIC in metasurfaces making them versatile candidate for low threshold lasing^29,30,92,94–96,200,201^. Lasing was reported from BIC-hosted optically pumped cavity at room temperature. It was shown that the width of lasing spectrum was broad at low pump power and turned to narrow lasing action at high pumping power. For robustness confirmation about 36 devices were fabricated for different sizes of metasurface arrays. Lasing was observed for different nanoresonator sizes and down to 8×8 array size. It was demonstrated that the lasing action is from BIC mode due to high Q-factor. The experimental results agree with theoretical calculations and lasing from different radii shows the scalability of the structure^29^. Another BIC based lasing was observed in GaAs nano-pillar, at high pump power and at temperature range of 77–200 K. It was shown that higher energy modes were excited at low Q-factor under the high pump power, and changing the temperature of device spectrally move the maximum of the gain at higher angles to lower threshold. Also, with variation in temperature the peak of the gain shifts to longer wavelengths (830–850 nm). Lasing spectra were achieved for four different cases (3 at 77 K and 1 at 200 K). The limitations remain in this structure is lasing at room temperature which was impossible due to low gain of GaAs because of high surface recombination^30^. They also reported lasing at room temperature from BIC in colloidal CdSe/CdZnS core-shell nano-platelets^94^. Etching and milling processes effect the gain due to additional scattering, the field trapping of BIC has been used to realize an etchless and highly controlled laser from a perovskite nanostructure^60^.

An ultra-low threshold of 12 µW, continuous wave from a miniaturized-BIC cavity by localizing light and carriers in three dimensions has been reported^93^. To achieve the continuous mode operations the size of cavity and lattice constant was tuned to match the frequency of cavity mode to the bandgap of heterostucture. Lasing from the 5 × 5 unit cells with a small mode volume also demonstrated. The heterostucture of square lattice array form a photonic crystal cavity, with a transition and boundary region. The structure demonstrates the emission spectrum from BIC with small pumping power. However, the fabrication imperfection slightly break the symmetry and produce split mode peak compared to theoretical calculations. Similarly, the Q-factor values are also deviated from the theoretical results. Although, the geometrical changes have no significant influence over the modes-position in the waveband. The wavelengths redshift due to decrease in number of unit cells which confirm that the lasing is from BIC. Periodic arrays comprising of few hundreds of meta-atoms are suitable for applications in passive optical devices^151,202^. In such devices the radiative and non radiative Q-factor deviation is due to certain limitations and imperfections of the structure, one of the common issues is scattering losses due to fabrication disorders. Secondly, the isolated BIC result in small Q-factor and after merging few BIC the Q-factor increases few orders of magnitude^202^.

Although, for lasing it is important to use a small array or finite structure critical for the spot size of the electrical or optical pumping. Due to reduced Q-factor of conventional BICs in the finite size of photonic crystal devices, some studies assumed the infinite size which driven the deviations of experimental results from computational. To address this issue lasing from symmetry protected, accidental and the combination of these two which makes super BIC was reported from one finite photonic structure^201^. They tuned the lattice constant to achieve lasing and super BIC. The radiative Q-factor remains as high as the experimental value of ~7300 compared to the conventional BICs in photonic devices. The enhanced Q-factor in the regime of super BIC results in significantly small threshold. Since lasing threshold reduced to 1.47 kW/cm^2^, is high compared to 12 µW reported in^93^.

#### Chiral emission and Brillouin zone folding

Owing to the high Q-factor mode, BIC as a topological state^203^ lying in the light continuum at a discrete position without radiation has been used for emissions in metasurface configurations. The emission of BIC structure radiated around the normal direction is bounded to the vortex beam^30,94,201^. Chiral BIC^77,89–91,152^ and photoluminescence from BIC inspired metasurfaces have been reported^87,88^, in the regime of Γ point through disturbing the configurations of the devices to generate a pair of circularly polarized states. Although, the two emitted lobes are still radiating near the normal direction as a consequence of weak directionality and spatially mixed in the far-field. A metasurface with square trapezoid holes has been reported to achieve high purity chiral emission^87^. However, the structure lakes the geometric tunability and a small deviations in configuration can disintegrate the BIC. Such approach is not extensible to practical devices with other materials due to its strict requirement of sophisticated fabrication methods. Recently, perovskite emission has been reported from symmetry protected BIC hosting structure^28^ as shown in Fig. 5a. The metasurface possesses various BICs, although the fundamental BIC is considered for spin valley locked emission. This TE_10_ (transverse electric-fundamental BIC) mode is spectrally isolated, the electric field lies inside the cylinder which are respectively favorable for lasing and occurring photoluminescence generation. At the center the magnetic field is out-of-plane and the electric field makes vortex around the periphery of the disk, this profile represents a Mei resonance of out-of-plane magnetic dipole. Since the BIC is completely decoupled from far-field radiations, so the Q-factor is up-to infinity and q-BIC has Giant finite Q-factor. The Brillouin zone folding replicate the valley of TE_10_ and result in a series of synthetic valleys in momentum space. The synthetic valleys are connected by translational symmetry with far-field emission. The fundamental valley Γ_0_ sustain the BIC and the eigen-polarization shows a vortex around BIC and the emission is weak in the vicinity of BIC. At the Γ_-1_ valley due to spin valley locking the eigen-polarizations at the center are left circularly polarized and the σ^-^ emission accordingly. At the Γ_+1_ valley exhibits the σ^+^ emission due to right circularly polarized eigen-polarization and inequivalent in emission to Γ_-1_ valley. A small shift of fundamental BIC to shorter wavelengths is due to notches because notches influence the dielectric constant of the cylindrical disk. The transverse magnetic mode (TM) is a Bloch mode which is not a BIC in un-notched metasurface, and different from TE, thus, cannot maintain lasing. Consequently, notches have no significant influence on this mode and low impact on the Brillouin zone folding.

Bloch surface waves has been used to enhance electric field confinement at the surface of dielectric substrate and the narrow resonances generated which strongly couples with monolayer TMD place at the top of gratings^138^. Strong Rabi splitting has been reported, which can boost the light matter interaction suitable for various optoelectronic applications (Fig. 5b).

#### Second harmonic generation

SHG is the most prominent nonlinear effect, in which the fundamental harmonic of a frequency can be non-linearly converted to its second harmonic with double frequency. GaAs and AlGaAs have been used as a constituent component for SHG in nonlinear metasurfaces due to its large bulk nonlinearity^203,204^. Recently, enhanced SHG was reported by coupling single layer TMD with a BIC-hosting structure. The structure possesses BIC and reducing the symmetry enhances the field localization in TMD layer which further result in boosted SHG^85^. As the transverse electric field (TE) is strongly localized within the wave-guide so, the coupling of monolayer with photonic BIC is not so efficient. Because, the monolayer TMD is placed at the surface and result in weak exciton-photon coupling. To strongly couple the monolayer with metasurface, Bloch surface waves (BSW) has been used to enhance the coupling, because in the BSW the electric field is confined at the surface of dielectric that strongly couples to the thin films at the surface^138^ (Fig. 5b).

#### Third and high harmonic generation

**Fig. 6:**
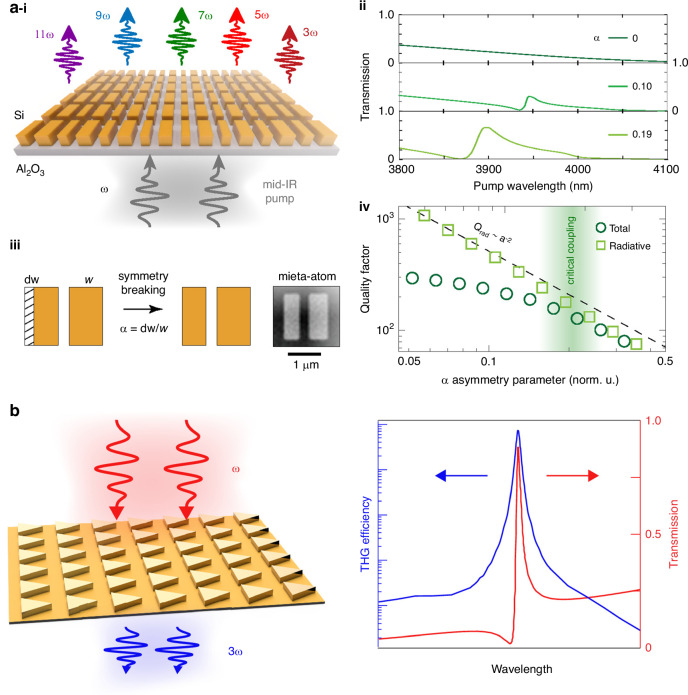
Nonlinear efficiencies from BIC-hosted metasurfaces. **a**–**i** Schematic of BIC hosting metasurface generating third to eleventh harmonics^98^. **ii** Transmission spectra with respect to pump wavelength^98^. **iii** Top view of unit cells with broken symmetry^98^, and **iv**. Q-factor as a function of asymmetry parameter values. Quasi-BIC appears with broken symmetry and high harmonic generation related to make best use of the electric field in the dielectric resonators. For resonant metasurface the nearfield enhancement is the interplay of non-radiative and radiative losses. The field enhancement is not maximum when the radiative losses are proportional to the coupling strength between mode and pump where the structure losses more energy. The field enhancement is maximum in critical coupling regime when non-radiative and radiative losses are comparable^98^. Reproduced with permissions^98^. Copyright © 2022, American Chemical Society. **b** THG reported from a BIC hosting metasurface and its control via asymmetry parameters. In this work it is shown that how the non-radiative and radiative losses control the THG intensity. The structure configurations have been tuned to critical coupling regime to exploit the maximum nonlinear frequency conversion efficiency^126^. Reproduced with permissions^126^. Copyright © 2019, American Chemical Society

It was uncovered that the harmonic generation intensity depends on the asymmetry of the structure. Using the critical coupling concept, THG was achieved by tuning the metasurface to critical coupling regime to reveal the effect of losses on the efficiency of nonlinear frequency conversion^126^ (see Fig. 6b). The THG efficiency is very high when the pump wavelength is tuned to q-BIC that compare to off-resonance wavelength. It is shown that the observed THG efficiency approaches the saturation with high pump power, although, it can damage the sample. The magnetic mode at q-BIC provides a dynamic control of the nonlinear emission efficiency. So, with tuning of pump excitation polarization condition, it was shown that an arrow-shaped image can be tune to visible through THG by adjusting the wavelength or polarization. Two opposite arrows are encoded through x-off and y-off centered shaped metasurfaces with excitation of x- and y-polarization (x-, 45°, and y-polarized pump). It was demonstrated that separate excitation of two q-BICs with two different polarizations is possible supported by two images. The dynamic control of the q-BIC spectral width can be achieved by tuning the dimensions of the structure and so the nonlinear images via modification in the wavelengths^52^. BIC has been discussed for applications high dimensional optical communication^80^. High harmonic generation have been reported from BIC modes in dielectric metasurfaces^97,98^ (Fig. 6ai–iv).

### Anapole state

#### Field enhancement

Anapole states result in strong near field inside the individual resonator structure with 3 times intensity enhancement of magnitude. It was shown that the peaks of field enhancement coincide with anapole mode and the enhancement factors are >500 and 1000. The disk with a narrow slot localized strong field compare to the solid one. This is because in the dielectric structures the electric field localization is mainly in the gaps and spaces between resonators. The displacement current is different in dielectrics (owing to bounded electrons due to phase retardation effects and field penetration) than metallic a structure in which conduction current is due to free electrons. For second order anapole mode the field enhancement is higher than first order anapole mode. It was shown that when the size of the slot is small, the near-field enhancement cannot interact with anapole state, and large size is also not favorable due to reverse direction of the electric field. For large field enhancement a certain size with a proper aspect ratio is important to obtain highest enhancement value. It was shown that intensity enhancement depends on the slot width, height, and diameter of the disk. The intensity increases with increase in height and diameter, however it decreases with increase in slot width^35^. The field enhancement reach to the highest in the dielectric-free region and make it as a subwavelength externally-accessible cavity, which can be exploited for microfluidic system to design high-efficiency biomedical, environmental monitoring, and for biological screening.

#### Lasing

The high-Q devices can be used for low-threshold lasing. A semiconductor based anapole hosting metasurface arranged in a thin ladder suspended in air demonstrate lasing at room temperature. The structure radiates a coherent beam with 2.2 nm narrow line-width and operating at low threshold. Due to the near-field of anapole mode a spontaneously polarized lasing has been reported from InGaAs nanodisk with generation of ultrafast pulses of 100 fs through mode locking few anapoles. Compared to classical nano-lasers the anapole based laser couples light as high as four orders of magnitude intensity to waveguide channels^31^.

#### Sensing

Metasurfaces possess excellent field confinement at nanoscale and enhanced light matter interactions. High sensitivity of their optical signatures to the nature of background medium attracted enormous attention for chemical and biological sensing applications. A plasmonic anapole with narrow line-width both numerically and experimentally demonstrate high sensitivity to perturb in background medium^73^. A reconfigurable sensor has been reported in terahertz metamaterials^205^ and metal-dielectric structure with anapole demonstrates enhanced sensitivity^206^.

#### Second and third harmonic generation

A bulk TMDs resonator was used to generate second harmonic despite their low nonlinearity in thick structures. Statistical analysis shows that SHG is very low in bulk TMDs. However, it is shown that resonantly enhanced SHG can be higher one order in magnitude than their monolayer counterparts if the unit-cell geometry of the nonlinear structure is carefully optimized^40,132^. A metal-dielectric hybrid structure was used to improve the nonlinear response. Adding the metallic portion to the dielectric structure enhance the nonlinear conversion efficiency by localizing strong electric field which support the improvement of anapole mode^68^. As the anapole modes can be achieved at a certain diameter/height ratio, this was observed in a germanium disk by variation in diameter. The diameter was varied at constant pump wavelength 1650 nm, the high THG values were achieved at anapole mode regime. The two electric dipoles result in an order smaller value of THG owing to low field confinement^68^. Self-boosting anapole and toroidal dipole enhanced THG in various configurations have been reported^43,114,168,207^.

## Summary and outlook

We discussed the recent advances in BIC and anapole modes for various nanophotonics applications. Material configurations used in recent past, different approaches and their applications in both linear and nonlinear systems are discussed. BIC and anapole modes can be achieved in plasmonic nanostructures with enhanced nearfield. However, the applications are limited due to material losses. In case of q-BIC, the plasmonic structure cannot sustain high Q-factor. Similarly, low nonlinearity in metallic structures limits plasmonic BIC and anapole modes for nonlinear applications. Still metallic nanostructures allow nonlinear effects to be used at low optical power, miniaturizing of the nonlinear components, and ultra-fast response time of excitation. The reduction in size contributes to develop integrated photonic devices and the fast response allows the signals to be controlled at femtoseconds.

In dielectric structures both BIC and anapole can be achieved with high Q-factor and field enhancement. Scattering due to rough surface, material losses, and fabrication disorder contributes to non-radiative losses. The field enhancement can be adjusted at a certain optimal ratio between the rates of different kind of losses. When the ratio is below the optimal value the non-radiative losses are high, thus, the structure outgoing energy is high, ultimately results in low field enhancement. The field enhancement is maximum, when the ratio is above the optimal value and the radiative losses become comparable to non-radiative. The field enhancement reaches the critical coupling regime when the rate of outgoing energy is lower than the incoming energy^98,100,208^. This is the criteria for harmonic generations to maximize the near-field inside the dielectric resonators. To manage the interplay between the losses symmetry reducing technique can be used to transform BIC to q-BIC, where the radiative Q-factor is proportional to the square of asymmetry parameter. On the other hand non-radiative losses limits the total Q-factor and its dependence is constant over the asymmetry and critical coupling can be realized at certain optimal value. The dielectric structures based metasurfaces can be used for various linear and nonlinear applications.

Anapole and BIC states have been discussed in bulk TMDs resonators. A well-pronounced dips in the far-field were achieved characterizing anapole modes. TMDs resonators support high SHG in well-defined geometry of the unit-cell compare to single layer TMDs. Statistically monolayer inherits high nonlinearity although bulk can support resonantly enhanced nonlinear efficiency. Few-layers TMDs support completely confined BIC states with Q-factor inclined to infinity at a discrete point. Owing to both optical modes and exciton effect the structure better sustain high Q-factor and well-pronounced optical modes in ideal BIC and q-BIC cases.

To further enhance the overall efficiency of the nanoscale structures one can design a hybrid combination of metals, dielectrics and TMDs. Monolayer TMDs support large values of SHG when coupled to asymmetric BIC-hosted structure. The q-BIC enhances the field in monolayer and boosts the SHG^85^. Such combinations provide enhancement and control of polaritons and nonlinear optical features in photonic devices due to its strong excitation interaction in TMD^136^. Metal-dielectric combinations have been discussed for prominent anapole and BIC modes. Recently, it was reported that the TE mode weakly excite the monolayer TMD due to its coupling to the dielectric gratings. Bloch surface wave are more suitable to enhance the coupling, achieve high Rabi splitting and high cooperativity^138^. In future, such multiphysics metasurfaces can fill the gaps and will improve the efficiencies of plasmonic and dielectric meta-optics.

BIC hosting structures have been used to demonstrate lasing at cryogenic and room temperatures. Low threshold lasing has been reported from the BIC structures in small array sizes. Fabrication imperfection is a challenge to avoid the split mode peak in experimental confirmation. In experiment, etching and milling processes affect the gain and incorporate additional scattering which influence the efficiency of the devices. A BIC hosting etchless structure has been used which provide an efficient controlled lasing action from a perovskite nanostructure. Recently, super BIC has been formed by merging accidental and symmetry protected BIC to further the efficiency. The desired Q-factor of a device is one of the main advantages of BIC, which is controlled by the driving parameter. Through Brillouin zone folding and array size tuning the Q-factor can be further improved.

TMDs can be fabricated as high refractive index dielectric metasurface which supports optical modes and exciton effect in ultra-thin geometries. At certain thickness the TMDs support Fabrey-Perot cavity which hybridizes with the exciton effect and can be termed as self-hybridized metasurfaces. Bulk TMD metasurfaces support both BIC and anapole modes and can be used to demonstrate high-Q optics in visible and part of infrared. To this end, only few research articles have been reported in the recent past. Van der Waals metasurfaces are still untouched and virgin area for various nanophotonic applications. Fabrication of few-layers TMD metasurface is a challenge, because few-layers TMD resonators are very thin and needs to be fabricated with sophisticated techniques.

The planar fabrication technology is suitable for fabricating metasurfaces and has advantages to translate laboratory work for industrial applications. Heavy investment of large corporations and aspiring start-up companies in this area witness a move to the commercial adoption. However, the incompatibility of materials used for metasurfaces, with industry standard is a challenge. For instance, noble metals are incompatible with fabrication of complementary metal-oxide semiconductor. Silicon based metasurfaces bridge this crack, although amorphous silicon facing process challenges, robustness and environmental variations. Advanced photo-masks are required for large area metasurface, and the existing electron beam lithography is time-consuming to pattern it. The control of angular, spectral dispersion and tunability of optical response is another challenge due to lack of unified approach to tune transmission amplitude, phase and polarization. The existing works focused on amplitude^209^ and phase modulation^210^. Due to requirement of simultaneous control of resonance position of two optical modes and parameters, no full polarization and continuous 2π-phase modulation has been demonstrated. However, change in the resonator materials by materials phase change, variations in geometric configurations and change in the background medium are the generic principles to induce the tunability according to the applications. (interested readers are referred to^211,212^).

We expect that the future work in nanophotonics will considerably extend the boundaries of BIC and anapole inspired metasurfaces to applications in various fields beyond up-to-the-minute progress. The organic and inorganic perovskite materials are emerging and interesting direction for light harvesting and optical devices by combining it with dielectric metasurfaces. Placing perovskite materials on the top or patterning into resonant metasurfaces leads to high light matter interaction. In recent past, anapole and BIC modes have been investigated in perovskite metasurfaces with enhanced Rabi splitting of around 250 meV in the coupling regime^28,59,84,213^. The multipolar toolbox of TMDs, dielectric and their combinations still have unforeseen effects that can be explored in future to improve the efficiency of the state-of-the art devices. The coupling of metasurfaces with other materials yields multiphysics metasurfaces, which are at their infancy and will further leads to novel phenomenon and regimes.
